# Refinement of the crystal structure of UOs_2_ from single-crystal X-ray diffraction data

**DOI:** 10.1107/S2414314626006917

**Published:** 2026-07-07

**Authors:** Sergei I. Ivlev, Matthias Conrad, Carsten Wolf, Florian Kraus

**Affiliations:** a Philipps-Universität Marburg, Fachbereich Chemie, Hans-Meerwein-Str. 4, 35032 Marburg, Germany; b Bruker AXS SE, Östliche Rheinbrückenstrasse 49, 76187 Karlsruhe, Germany; c Anorganische Chemie, Fluorchemie, Institut für Anorganische Chemie, Universität Bonn, Gerhard-Domagk-Strasse 1, 53121 Bonn, Germany; Vienna University of Technology, Austria

**Keywords:** crystal structure, inter­metallics, single-crystal X-ray diffraction, redetermination, MgCu_2_ structure type

## Abstract

Single crystals of uranium diosmium, UOs_2_, were obtained during an investigation of the uranium-osmium binary system by an inter­action of uranium metal with osmium metal in an arc furnace and subsequent annealing. The refinement of the crystal structure of UO_2_ was carried out for the first time using single-crystal X-ray diffraction data. In contrast to previous structure reports based on powder diffraction data, the U and Os atoms were refined with all *U^ij^* terms of the displacement parameters considered. UOs_2_ adopts the MgCu_2_ structure type.

## Abstract 2

Refinement of the crystal structure of UOs_2_ was performed for the first time by using single-crystal X-ray diffraction data, which allowed the refinement of all *U^ij^* terms of the displacement parameters.

## Graphical abstract

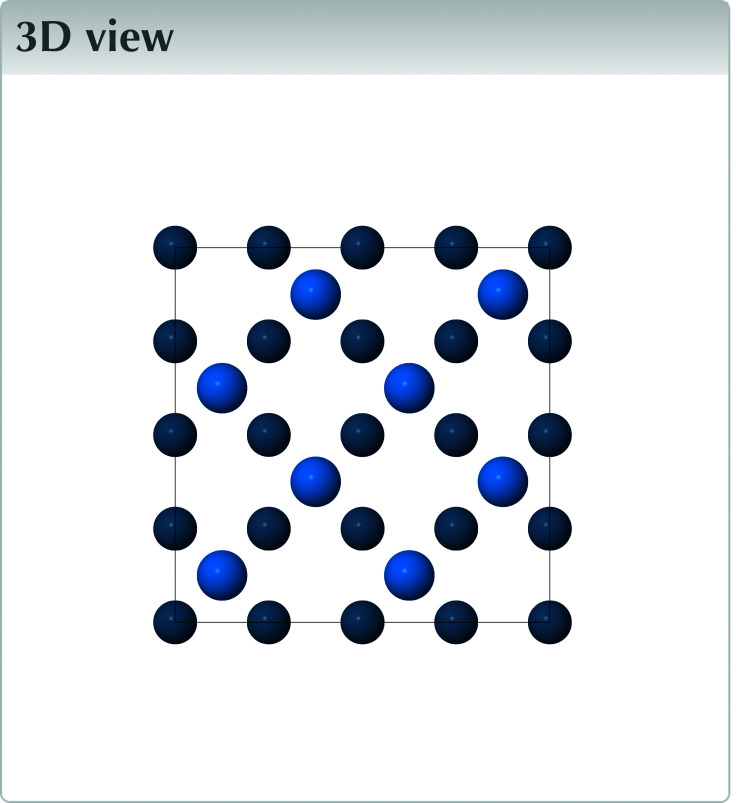

## Structure description

**Figure 1:**
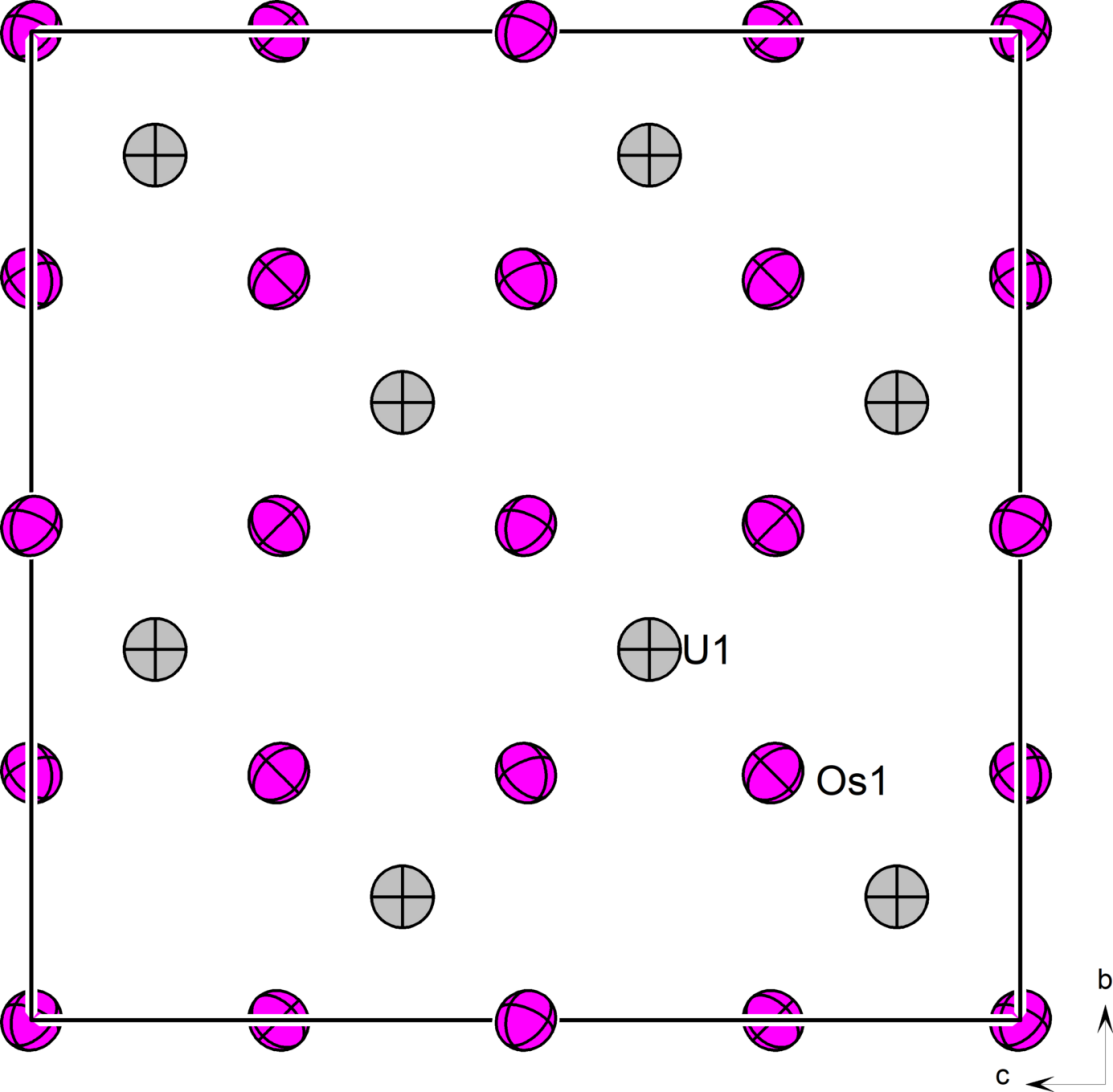
Crystal structure of UOs_2_ in a projection along [100]. Displacement ellipsoids are shown at the 90% probability level.

According to the Inorganic Crystal Structure Database (ICSD; Zagorac *et al.*, 2019 ▸), the first crystal structure determination of the binary phase UOs_2_ was carried out by Heal & Williams (1955 ▸) using a powder sample and a diffractometer operated with Co radiation. They reported the compound to crystallize in the space group *Fd*
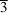
*m* (No. 227) with *a* = 7.5125 (5) Å, *V* = 423.99 Å^3^, *Z* = 8 at 297 K belonging to the MgCu_2_ structure type. Subsequent structure reports confirmed this finding with the unit-cell parameter to be in very close agreement with the one originally reported: 7.514 Å [Knapton, 1963 ▸; room-temperature data, no standard uncertainty (s.u.) given]; 7.501 (4) Å (Holleck *et al.*, 1975 ▸; room-temperature data); 7.514 Å (Mentink *et al.*, 1992 ▸; room-temperature data, no s.u. given); 7.4919 (Nishioka *et al.*, 1994 ▸; room-temperature data, no s.u. given). Nishioka *et al.* (1994 ▸) also reported the existence of a polymorph of UOs_2_ belonging to the MgZn_2_ structure type [space group *P*6_3_/*mmc* (No. 194) with *a* = 5.49, *c* = 8.71 Å, *V* = 227.35 Å^3^, *Z* = 4; room temperature data, no s.u. given]. All mentioned crystal-structure refinements were carried out on the basis of powder diffraction techniques with both Os and U atoms being refined isotropically. Although an anisotropic refinement of the U atom is equivalent to the isotropic one due to symmetry constraints, the site symmetry of the Os atom does allow non-zero off-diagonal *U^ij^* terms, when refined anisotropically. Here we report our results on the crystal-structure determination of UOs_2_ using single-crystal X-ray diffraction data at 100 K that allowed to use the anisotropic approximation for the displace­ment parameters (Fig. 1 ▸).

The unit-cell parameter of UOs_2_ obtained from the single-crystal diffraction experiment (Table 1 ▸) is in good agreement with all previously reported data. The U atom resides on the special 8*a* (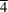
3*m*) Wyckoff position. The Os atom occupies the special 16*d* (.
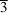
*m*) Wyckoff position. The closest inter­atomic distances are U⋯U 3.24669 (8), Os⋯Os 2.65091 (7), and U⋯Os 3.10847 (8) Å.

Since UOs_2_ is isotypic to the well known Laves phase MgCu_2_, its crystal structure will only be described very briefly. The U atoms are arranged like the atoms in the cubic diamond structure type. The Os atoms form Os_4_ tetra­hedra. Their virtual centres of gravity are also arranged according to the cubic diamond structure type, and the two networks inter­penetrate. Overall, the coordination number of the Os atom is 12 within a slightly distorted icosa­hedral coordination environment. Each Os atom resides within the centre of a U_6_ ring adopting a chair conformation and is additionally surrounded by six Os atoms in the shape of a trigonal anti­prism. Overall, the coordination number of the U atom is 16. Each U atom is surrounded tetra­hedrally by four U atoms and by twelve Os atoms in the shape of a fourfold truncated tetra­hedron, which is sometimes referred to as the Friauf polyhedron (Wells, 1975 ▸).

## Synthesis and crystallization

Single crystals of UOs_2_ were obtained by a direct reaction between stoichiometric amounts of uranium and osmium metal powders in an arc furnace. The polycrystalline reaction product was subsequently annealed in a tantalum ampule at 1073 K for 11 d and crushed in oil under a microscope to select a single crystal suitable for the diffraction study.

## Refinement

Details of data collection and structure refinement are given in Table 1 ▸.

## Supplementary Material

Crystal structure: contains datablock(s) I. DOI: 10.1107/S2414314626006917/wm4251sup1.cif

Structure factors: contains datablock(s) I. DOI: 10.1107/S2414314626006917/wm4251Isup2.hkl

CCDC reference: 2567529

Additional supporting information:  crystallographic information; 3D view; checkCIF report

## Figures and Tables

**Table 1:** Experimental details

Crystal data	
Chemical formula	UOs_2_
*M*_r_	618.43
Crystal system, space group	Cubic, *Fd* 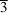 *m*
Temperature (K)	100
*a* (Å)	7.4979 (2)
*V* (Å^3^)	421.52 (3)
*Z*	8
Radiation type	Mo *K*α
μ (mm^−1^)	196.43
Crystal size (mm)	0.05 × 0.04 × 0.03

Data collection	
Diffractometer	Bruker D8 VENTURE
Absorption correction	Numerical (*SADABS*; Krause *et al.*, 2015 ▸)
*T*_min_, *T*_max_	0.014, 0.127
No. of measured, independent and observed [*I* > 2σ(*I*)] reflections	2221, 69, 66
*R*_int_	0.083
(sin θ/λ)_max_ (Å^−1^)	0.830

Refinement	
*R*[*F*^2^ > 2σ(*F*^2^)], *wR*(*F*^2^), *S*	0.020, 0.047, 1.40
No. of reflections	69
No. of parameters	5
Δρ_max_, Δρ_min_ (e Å^−3^)	1.92, −2.16

Computer programs: *APEX5* (Bruker, 2023 ▸), *SAINT* (Bruker, 2019 ▸), *SHELXT* (Sheldrick, 2015*a* ▸), *SHELXL* (Sheldrick, 2015*b* ▸), *DIAMOND* (Brandenburg, 2022 ▸) and *publCIF* (Westrip, 2010 ▸).

## full crystallographic data

### Uranium diosmium Crystal data

**Table:**

UOs_2_	Mo *K*α radiation, λ = 0.71073 Å
*M**_r_* = 618.43	Cell parameters from 2037 reflections
Cubic, *F**d*3*m*	θ = 4.7–36.2°
*a* = 7.4979 (2) Å	µ = 196.43 mm^−^^1^
*V* = 421.52 (3) Å^3^	*T* = 100 K
*Z* = 8	Block, metallic grey
*F*(000) = 1952	0.05 × 0.04 × 0.03 mm
*D*_x_ = 19.490 Mg m^−^^3^	

### Uranium diosmium Data collection

**Table:**

Bruker D8 VENTURE diffractometer	66 reflections with *I* > 2σ(*I*)
Radiation source: microfocus X-ray tube	*R*_int_ = 0.083
ω and φ scans	θ_max_ = 36.2°, θ_min_ = 4.7°
Absorption correction: numerical (*SADABS*; Krause *et al.*, 2015)	*h* = −11→10
*T*_min_ = 0.014, *T*_max_ = 0.127	*k* = −10→11
2221 measured reflections	*l* = −12→10
69 independent reflections	

### Uranium diosmium Refinement

**Table:**

Refinement on *F*^2^	Primary atom site location: dual
Least-squares matrix: full	Secondary atom site location: difference Fourier map
*R*[*F*^2^ > 2σ(*F*^2^)] = 0.020	*w* = 1/[σ^2^(*F*_o_^2^) + (0.0161*P*)^2^ + 10.8153*P*] where *P* = (*F*_o_^2^ + 2*F*_c_^2^)/3
*wR*(*F*^2^) = 0.047	(Δ/σ)_max_ < 0.001
*S* = 1.40	Δρ_max_ = 1.92 e Å^−^^3^
69 reflections	Δρ_min_ = −2.16 e Å^−^^3^
5 parameters	Extinction correction: SHELXL2019/2 (Sheldrick 2015b), Fc^*^=kFc[1+0.001xFc^2^λ^3^/sin(2θ)]^-1/4^
0 restraints	Extinction coefficient: 0.00055 (10)

### Uranium diosmium Special details

**Table:**

Geometry. All esds (except the esd in the dihedral angle between two l.s. planes) are estimated using the full covariance matrix. The cell esds are taken into account individually in the estimation of esds in distances, angles and torsion angles; correlations between esds in cell parameters are only used when they are defined by crystal symmetry. An approximate (isotropic) treatment of cell esds is used for estimating esds involving l.s. planes.

### Uranium diosmium Fractional atomic coordinates and isotropic or equivalent isotropic displacement parameters (Å^2^)

**Table:**

	*x*	*y*	*z*	*U*_iso_*/*U*_eq_	
U1	0.875000	0.375000	0.375000	0.0088 (3)	
Os1	0.500000	0.250000	0.250000	0.0082 (3)	

### Uranium diosmium Atomic displacement parameters (Å^2^)

**Table:**

	*U*^11^	*U*^22^	*U*^33^	*U*^12^	*U*^13^	*U*^23^
U1	0.0088 (3)	0.0088 (3)	0.0088 (3)	0.000	0.000	0.000
Os1	0.0082 (3)	0.0082 (3)	0.0082 (3)	0.00062 (12)	0.00062 (12)	−0.00062 (12)

### Uranium diosmium Geometric parameters (Å, º)

**Table:**

U1—Os1^i^	3.1085 (1)	U1—Os1^xi^	3.1085 (1)
U1—Os1	3.1085 (1)	U1—U1^xii^	3.2467 (1)
U1—Os1^ii^	3.1085 (1)	U1—U1^xiii^	3.2467 (1)
U1—Os1^iii^	3.1085 (1)	U1—U1^xiv^	3.2467 (1)
U1—Os1^iv^	3.1085 (1)	U1—U1^xv^	3.2467 (1)
U1—Os1^v^	3.1085 (1)	Os1—Os1^xvi^	2.6509 (1)
U1—Os1^vi^	3.1085 (1)	Os1—Os1^ix^	2.6509 (1)
U1—Os1^vii^	3.1085 (1)	Os1—Os1^xvii^	2.6509 (1)
U1—Os1^viii^	3.1085 (1)	Os1—Os1^i^	2.6509 (1)
U1—Os1^ix^	3.1085 (1)	Os1—Os1^ii^	2.6509 (1)
U1—Os1^x^	3.1085 (1)	Os1—Os1^xviii^	2.6509 (1)
			
Os1^i^—U1—Os1	50.5	Os1^ii^—U1—U1^xiv^	58.5
Os1^i^—U1—Os1^ii^	50.5	Os1^iii^—U1—U1^xiv^	150.5
Os1—U1—Os1^ii^	50.5	Os1^iv^—U1—U1^xiv^	58.518 (1)
Os1^i^—U1—Os1^iii^	144.9	Os1^v^—U1—U1^xiv^	100.025 (1)
Os1—U1—Os1^iii^	95.2	Os1^vi^—U1—U1^xiv^	100.025 (1)
Os1^ii^—U1—Os1^iii^	117.0	Os1^vii^—U1—U1^xiv^	58.518 (1)
Os1^i^—U1—Os1^iv^	95.2	Os1^viii^—U1—U1^xiv^	150.5
Os1—U1—Os1^iv^	144.9	Os1^ix^—U1—U1^xiv^	150.5
Os1^ii^—U1—Os1^iv^	117.0	Os1^x^—U1—U1^xiv^	58.5
Os1^iii^—U1—Os1^iv^	117.0	Os1^xi^—U1—U1^xiv^	58.5
Os1^i^—U1—Os1^v^	95.2	U1^xii^—U1—U1^xiv^	109.471 (1)
Os1—U1—Os1^v^	117.0	U1^xiii^—U1—U1^xiv^	109.5
Os1^ii^—U1—Os1^v^	144.9	Os1^i^—U1—U1^xv^	58.5
Os1^iii^—U1—Os1^v^	95.2	Os1—U1—U1^xv^	58.5
Os1^iv^—U1—Os1^v^	50.5	Os1^ii^—U1—U1^xv^	100.0
Os1^i^—U1—Os1^vi^	144.9	Os1^iii^—U1—U1^xv^	100.0
Os1—U1—Os1^vi^	117.0	Os1^iv^—U1—U1^xv^	100.025 (1)
Os1^ii^—U1—Os1^vi^	95.2	Os1^v^—U1—U1^xv^	58.5
Os1^iii^—U1—Os1^vi^	50.5	Os1^vi^—U1—U1^xv^	150.5
Os1^iv^—U1—Os1^vi^	95.2	Os1^vii^—U1—U1^xv^	150.5
Os1^v^—U1—Os1^vi^	117.0	Os1^viii^—U1—U1^xv^	58.5
Os1^i^—U1—Os1^vii^	117.0	Os1^ix^—U1—U1^xv^	58.5
Os1—U1—Os1^vii^	144.9	Os1^x^—U1—U1^xv^	150.5
Os1^ii^—U1—Os1^vii^	95.2	Os1^xi^—U1—U1^xv^	58.5
Os1^iii^—U1—Os1^vii^	95.2	U1^xii^—U1—U1^xv^	109.471 (1)
Os1^iv^—U1—Os1^vii^	50.5	U1^xiii^—U1—U1^xv^	109.5
Os1^v^—U1—Os1^vii^	95.2	U1^xiv^—U1—U1^xv^	109.5
Os1^vi^—U1—Os1^vii^	50.5	Os1^xvi^—Os1—Os1^ix^	180.0
Os1^i^—U1—Os1^viii^	117.0	Os1^xvi^—Os1—Os1^xvii^	120.0
Os1—U1—Os1^viii^	95.2	Os1^ix^—Os1—Os1^xvii^	60.0
Os1^ii^—U1—Os1^viii^	144.9	Os1^xvi^—Os1—Os1^i^	60.0
Os1^iii^—U1—Os1^viii^	50.5	Os1^ix^—Os1—Os1^i^	120.0
Os1^iv^—U1—Os1^viii^	95.2	Os1^xvii^—Os1—Os1^i^	180.0
Os1^v^—U1—Os1^viii^	50.5	Os1^xvi^—Os1—Os1^ii^	60.0
Os1^vi^—U1—Os1^viii^	95.2	Os1^ix^—Os1—Os1^ii^	120.0
Os1^vii^—U1—Os1^viii^	117.0	Os1^xvii^—Os1—Os1^ii^	120.0
Os1^i^—U1—Os1^ix^	95.2	Os1^i^—Os1—Os1^ii^	60.0
Os1—U1—Os1^ix^	50.5	Os1^xvi^—Os1—Os1^xviii^	120.0
Os1^ii^—U1—Os1^ix^	95.2	Os1^ix^—Os1—Os1^xviii^	60.0
Os1^iii^—U1—Os1^ix^	50.5	Os1^xvii^—Os1—Os1^xviii^	60.0
Os1^iv^—U1—Os1^ix^	144.9	Os1^i^—Os1—Os1^xviii^	120.0
Os1^v^—U1—Os1^ix^	95.2	Os1^ii^—Os1—Os1^xviii^	180.0
Os1^vi^—U1—Os1^ix^	95.2	Os1^xvi^—Os1—U1	115.2
Os1^vii^—U1—Os1^ix^	144.9	Os1^ix^—Os1—U1	64.8
Os1^viii^—U1—Os1^ix^	50.5	Os1^xvii^—Os1—U1	115.2
Os1^i^—U1—Os1^x^	95.216 (1)	Os1^i^—Os1—U1	64.8
Os1—U1—Os1^x^	95.2	Os1^ii^—Os1—U1	64.8
Os1^ii^—U1—Os1^x^	50.5	Os1^xviii^—Os1—U1	115.2
Os1^iii^—U1—Os1^x^	95.2	Os1^xvi^—Os1—U1^xix^	64.8
Os1^iv^—U1—Os1^x^	95.2	Os1^ix^—Os1—U1^xix^	115.2
Os1^v^—U1—Os1^x^	144.9	Os1^xvii^—Os1—U1^xix^	64.8
Os1^vi^—U1—Os1^x^	50.5	Os1^i^—Os1—U1^xix^	115.2
Os1^vii^—U1—Os1^x^	50.5	Os1^ii^—Os1—U1^xix^	115.2
Os1^viii^—U1—Os1^x^	144.9	Os1^xviii^—Os1—U1^xix^	64.8
Os1^ix^—U1—Os1^x^	117.0	U1—Os1—U1^xix^	180.0
Os1^i^—U1—Os1^xi^	50.5	Os1^xvi^—Os1—U1^xv^	115.2
Os1—U1—Os1^xi^	95.2	Os1^ix^—Os1—U1^xv^	64.8
Os1^ii^—U1—Os1^xi^	95.216 (1)	Os1^xvii^—Os1—U1^xv^	115.2
Os1^iii^—U1—Os1^xi^	144.9	Os1^i^—Os1—U1^xv^	64.8
Os1^iv^—U1—Os1^xi^	50.5	Os1^ii^—Os1—U1^xv^	115.2
Os1^v^—U1—Os1^xi^	50.5	Os1^xviii^—Os1—U1^xv^	64.8
Os1^vi^—U1—Os1^xi^	144.9	U1—Os1—U1^xv^	63.0
Os1^vii^—U1—Os1^xi^	95.2	U1^xix^—Os1—U1^xv^	117.0
Os1^viii^—U1—Os1^xi^	95.2	Os1^xvi^—Os1—U1^xx^	64.8
Os1^ix^—U1—Os1^xi^	117.0	Os1^ix^—Os1—U1^xx^	115.239 (1)
Os1^x^—U1—Os1^xi^	117.0	Os1^xvii^—Os1—U1^xx^	64.8
Os1^i^—U1—U1^xii^	150.5	Os1^i^—Os1—U1^xx^	115.2
Os1—U1—U1^xii^	150.5	Os1^ii^—Os1—U1^xx^	64.8
Os1^ii^—U1—U1^xii^	150.5	Os1^xviii^—Os1—U1^xx^	115.2
Os1^iii^—U1—U1^xii^	58.5	U1—Os1—U1^xx^	117.0
Os1^iv^—U1—U1^xii^	58.5	U1^xix^—Os1—U1^xx^	63.0
Os1^v^—U1—U1^xii^	58.5	U1^xv^—Os1—U1^xx^	180.0
Os1^vi^—U1—U1^xii^	58.5	Os1^xvi^—Os1—U1^xxi^	64.8
Os1^vii^—U1—U1^xii^	58.5	Os1^ix^—Os1—U1^xxi^	115.2
Os1^viii^—U1—U1^xii^	58.5	Os1^xvii^—Os1—U1^xxi^	115.2
Os1^ix^—U1—U1^xii^	100.025 (1)	Os1^i^—Os1—U1^xxi^	64.8
Os1^x^—U1—U1^xii^	100.0	Os1^ii^—Os1—U1^xxi^	115.2
Os1^xi^—U1—U1^xii^	100.0	Os1^xviii^—Os1—U1^xxi^	64.8
Os1^i^—U1—U1^xiii^	100.0	U1—Os1—U1^xxi^	117.0
Os1—U1—U1^xiii^	58.518 (1)	U1^xix^—Os1—U1^xxi^	63.0
Os1^ii^—U1—U1^xiii^	58.518 (1)	U1^xv^—Os1—U1^xxi^	63.0
Os1^iii^—U1—U1^xiii^	58.5	U1^xx^—Os1—U1^xxi^	117.0
Os1^iv^—U1—U1^xiii^	150.5	Os1^xvi^—Os1—U1^xiii^	115.2
Os1^v^—U1—U1^xiii^	150.5	Os1^ix^—Os1—U1^xiii^	64.8
Os1^vi^—U1—U1^xiii^	58.5	Os1^xvii^—Os1—U1^xiii^	64.8
Os1^vii^—U1—U1^xiii^	100.0	Os1^i^—Os1—U1^xiii^	115.2
Os1^viii^—U1—U1^xiii^	100.0	Os1^ii^—Os1—U1^xiii^	64.8
Os1^ix^—U1—U1^xiii^	58.5	Os1^xviii^—Os1—U1^xiii^	115.2
Os1^x^—U1—U1^xiii^	58.5	U1—Os1—U1^xiii^	63.0
Os1^xi^—U1—U1^xiii^	150.5	U1^xix^—Os1—U1^xiii^	117.0
U1^xii^—U1—U1^xiii^	109.471 (1)	U1^xv^—Os1—U1^xiii^	117.0
Os1^i^—U1—U1^xiv^	58.5	U1^xx^—Os1—U1^xiii^	63.0
Os1—U1—U1^xiv^	100.0	U1^xxi^—Os1—U1^xiii^	180.0

Symmetry codes: (i) −*x*+5/4, *y*, −*z*+1/4; (ii) −*x*+5/4, −*y*+1/4, *z*; (iii) −*x*+5/4, −*y*+3/4, *z*+1/2; (iv) −*x*+7/4, −*y*+3/4, *z*; (v) *x*+1/2, *y*+1/2, *z*; (vi) *x*+1/2, *y*, *z*+1/2; (vii) −*x*+7/4, *y*, −*z*+3/4; (viii) −*x*+5/4, *y*+1/2, −*z*+3/4; (ix) *x*, −*y*+3/4, −*z*+3/4; (x) *x*+1/2, −*y*+1/4, −*z*+3/4; (xi) *x*+1/2, −*y*+3/4, −*z*+1/4; (xii) −*x*+2, −*y*+1, −*z*+1; (xiii) −*y*+1, *x*−3/4, *z*+1/4; (xiv) −*x*+2, −*y*+1/2, −*z*+1/2; (xv) −*x*+3/2, −*y*+1, −*z*+1/2; (xvi) *x*, −*y*+1/4, −*z*+1/4; (xvii) −*x*+3/4, *y*, −*z*+3/4; (xviii) −*x*+3/4, −*y*+3/4, *z*; (xix) −*x*+1, −*y*+1/2, −*z*+1/2; (xx) *x*−1/2, *y*−1/2, *z*; (xxi) *x*−1/2, *y*, *z*−1/2.
